# Quantitative Measurement of the Magnetic Moment of Individual Magnetic Nanoparticles by Magnetic Force Microscopy

**DOI:** 10.1002/smll.201200420

**Published:** 2012-06-22

**Authors:** Sibylle Sievers, Kai-Felix Braun, Dietmar Eberbeck, Stefan Gustafsson, Eva Olsson, Hans Werner Schumacher, Uwe Siegner

**Affiliations:** 1 Physikalisch-Technische BundesanstaltBundesallee 100, 38116 Braunschweig, Germany; 2 Physikalisch-Technische BundesanstaltAbbestraße 2-12, 10587 Berlin, Germany; 3 Department of Applied Physics, Chalmers University of Technology41296 Gothenburg, Sweden

**Keywords:** calibration, magnetic force microscopy, magnetic properties, nanoparticles, paramagnetism

## Abstract

The quantitative measurement of the magnetization of individual magnetic nanoparticles (MNPs) using magnetic force microscopy (MFM) is described. Quantitative measurement is realized by calibration of the MFM signal using an MNP reference sample with traceably determined magnetization. A resolution of the magnetic moment of the order of 10^−18^ A m^2^ under ambient conditions is demonstrated, which is presently limited by the tip's magnetic moment and the noise level of the instrument. The calibration scheme can be applied to practically any magnetic force microscope and tip, thus allowing a wide range of future applications, for example in nanomagnetism and biotechnology.

## 1. Introduction

Magnetic nanoparticles (MNPs) show potential use in a wide range of applications, for example, in biomedicine and data storage.^1–4^ For research purposes as well as for quality control, a precise characterization of the magnetic properties of the MNPs is essential. However, standard characterization techniques such as superconducting quantum interference device (SQUID) magnetometry only allow the measurement of integral properties of ensembles of MNPs. The direct characterization of individual particles is only possible by microscopy techniques. Due to its high spatial resolution, magnetic force microscopy (MFM) is a powerful tool for imaging magnetic nanostructures. MFM is a stray-field-sensitive technique with a resolution down to 10 nm. The ability of MFM to detect superparamagnetic and low-coercivity MNPs and the interpretation of the resulting MFM images are subjects of ongoing research.^5–10^ Proksch et al. presented the quantitative measurement of the magnetic moment of a chain of MNPs contained in isolated magnetotactic bacteria.^11^ The analysis was done by fitting an MFM tip model that is the result of a complex analysis of the particular tip to the dipolar signal from the magnetotactic bacteria. However, a model-independent technique allowing quantitative analysis of the measured MFM data of individual MNPs with respect to the magnetic moment would be useful but is lacking. The standard approach for the quantitative characterization of small structures is the point probe approximation.^12–14^ However, the point probe approach disregards the nonlocal character of the MFM tip magnetization and, therefore, is inadequate for patterns with dimensions comparable to the tip dimensions.

Herein, it is shown that a calibration of arbitrary MFM tips can be obtained that allows the direct quantitative measurement of the magnetic moment of spherical nanoparticles without a priori statements on the tip properties. No assumption regarding the tip geometry is required since the stray field of a homogeneously magnetized sphere equals the stray field of a point dipole positioned in the center of the MNP, that is, the functional form of the stray field does not depend on the diameter of the MNP. This calibration scheme is based on an MNP reference sample, which provides traceability to the SI units for the measurement of magnetic moments of individual MNPs as small as 10^−18^ A m^2^.

## 2. Theory

In MFM, the tip scans over the sample at a given lift height *h* and the frequency shift Δ*f* of the oscillating MFM cantilever is recorded. The frequency shift Δ*f* can be calculated from the force **F** that is acting on the magnetic tip in the stray field **H** of the sample as 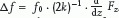.^15^ Here, *k* and *f*_0_ are the spring constant and resonance frequency of the free cantilever, respectively. *F*_z_ is the component of **F** perpendicular to the sample surface. Since 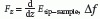, Δ*f* can also be expressed as 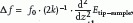, where *E*_tip–sample_ is the interaction energy between the magnetic stray field of the MNP and the tip. *E*_tip–sample_ can be expressed in terms of a convolution of the tip magnetization **M**_tip_ and the sample stray field **H**, which reads for a tip whose apex is at the position **r** = (*x*,*y*,*z*):

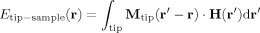
(1)

Now, we focus on single-domain MNPs. In good approximation, these can be modeled as magnetic nanospheres with saturation magnetization *M*_S_ and volume 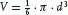, with *d* being the diameter of the MNP. For this geometry the stray field **H** is equal to the stray field of a magnetic dipole that is positioned in the center of the sphere.^16^ The absolute value *m* of the dipole moment **m** is then given by 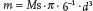 and the stray field of an MNP that is located at **r′** = 0 is given by:

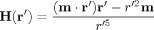
(2)

**Figure 1:**
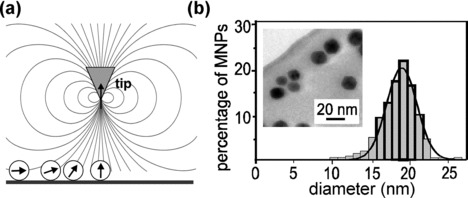
a) The particle's magnetic moment is aligned with the local magnetic field below the tip. b) The inset shows a TEM picture of MNPs of the sample SHP20. The plot shows the core diameter distribution estimated from the TEM image.

If the magnetic anisotropy of a nanoparticle is sufficiently small, the stray field emerging from the magnetic tip is sufficient to fully align the nanoparticle magnetization as sketched in **Figure** **1**a.^10^ Since for the most common MFM tips the stray field underneath the tip is oriented perpendicular to the *x*–*y* scanning plane, the magnetization of the nanoparticle is also aligned perpendicular when the MFM tip is located at a lift height *h* above the center position of the nanoparticle, that is, at *r* = (0, 0, *z*) = (0, 0, *h*). At this specific position, the particle magnetization **m** is given by **m** = *m*·**z**, with **z** the unit vector in the *z* direction. Hence, the frequency shift Δ*f* over the center of the MNP can be calculated as:

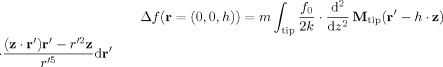
(3)

The integral term becomes a constant that only depends on the magnetic properties of the magnetic probe. For a given tip height *h* it therefore represents a tip-dependent proportionality constant *c*(*h*)^−1^ connecting the magnetic moment *m* of the spherical nanoparticle and the measured MFM frequency shift by 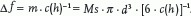. Note that over the center of each MNP, when the particle magnetization is aligned vertically, the frequency shift Δ*f*_max_ detected for each particle will reach its maximum value. As a consequence, for spherical MNPs a calibration of the magnetic tip can be achieved by measuring the maxima of the frequency shift, Δ*f*_max_*,* for a given lift height of a set of nanoparticles with known magnetic moment.

## 3. Experimental Details

In the following, an example of a calibration is discussed, which clarifies the technical details and verifies the feasibility of the calibration approach. The MFM calibration is based on a MNP reference sample that has to fulfill the following requirements: 1) the MNPs do not agglomerate and 2) the magnetization of the MNPs is well known. We selected commercial magnetite nanoparticles with 20 nm nominal diameter, in the following referred to as SHP20.^17^ A sample of well-separated MNPs was prepared by pouring the particles in solution onto a silicon substrate that was exposed to a vertical magnetic field (≍500 mT). Thereby the particles were magnetically aligned and repelled each other, which prevented particle agglomeration during drying.

The MNPs' size distribution was determined by transmission electron microscopy (TEM; Figure 1b). The resulting mean particle diameter is *d*_TEM_ = (18.7 ± 3) nm. To traceably determine the saturation magnetization *M*_S_ of the reference MNPs, the total magnetic moment of a small sample volume of the MNP suspension was measured by SQUID magnetometry. The iron content and hence the volume of magnetite in the sample was determined by titration using Prussian blue staining. From these data the saturation magnetization was determined to be *M*_S_ = (250 ± 10) kA m^−1^ at 293 K. The measured value of *M*_S_ allows a calculation of the magnetic moment of the SHP20 MNPs for a given particle diameter *d* using the relation, 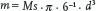 assuming a homogeneous distribution of magnetic material over the whole volume of the particle. This can be reasonably assumed, since magnetite is the most stable iron oxide.

**Figure 2:**
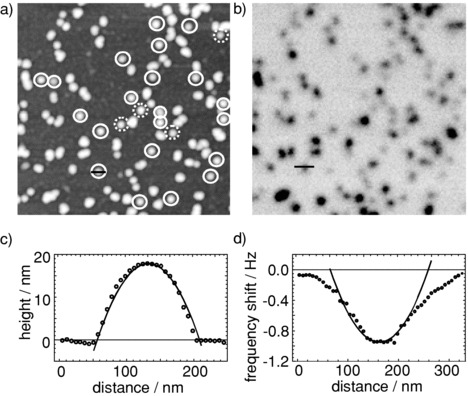
a) AFM image and b) the corresponding MFM image recorded at a lift height of 50 nm above the substrate of sample SHP20. The sample area is 3.4 × 3.4 μm^2^. c,d) Line scans across a nanoparticle of topography (c) and of relative frequency shift (d) (see text).

A SHP20 reference sample, prepared as described above, was employed to calibrate the signal of commercial MFM cantilevers.^18^ Atomic force microscopy (AFM) and MFM were performed using a commercial MFM instrument.^19^ The substrate surface plane was determined by fitting a plane to the measured topography data from substrate areas without MNPs. The MFM image was taken in a self-excitation mode and the tip scanned the sample at a constant lift height *h* with respect to the calculated substrate surface plane. The MFM instrument was operated with a closed-loop scanner to reduce the effect of piezo drift to the maximum extent. In the first step an AFM topography image was recorded, as shown in **Figure** **2**a. Among the large number of particles visible in the image, 25 separated nanoparticles (marked by circles) could be identified; all the other particles are clusters. Clusters of MNPs could be identified by having a nonrotational symmetric shape in the AFM image. These clusters could not be employed for tip calibration since the point dipole approximation does not hold for the cluster stray field. Out of the 25 separated nanoparticles 21 (continuous circles in Figure 2a) were used for the MFM calibration. For the others (dotted circles), the MFM signal was overlaid by the signal of neighboring clusters. Again this could be identified by a lack of rotational symmetry of the MFM signal of the MNPs.

## 4. Results

### 4.1. Determination of the Calibration Factor

The height and thus the diameter *d* of the particles was determined by fitting a two-dimensional parabolic function to the area in the AFM image near the maximum height of each MNP (Figure 2c). The fitting is used to tackle the problem of noise in the AFM image. As the tip shape and thus the theoretical function of the AFM signal for an MNP are unknown, a simple two-dimensional parabolic shape was found to be suitable to approximate the data. This approach results in a good approximation of the measurement data (see Figure 2c) and hence in a precise determination of *d*. The substrate level was determined by fitting a plane to nanoparticle-free substrate areas. For the AFM image shown in Figure 2a the resulting mean particle diameter is *d*_AFM_ = (16.9 ± 2.9) nm, in good agreement with the TEM analysis. In solution, the SHP20 nanoparticles are coated with oleic acid and amphiphilic polymer. However, this shell shrinks during the drying process. Organic coatings are not visible in TEM characterization and TEM only measures the inorganic core size. The good agreement between the results of both techniques shows that the coating has no significant influence on the diameter measured by AFM. From this we assume that AFM essentially measures the diameter of the magnetic core.

In a second step, the corresponding MFM image was taken at a constant lift height of *h* = 50 nm above the substrate (Figure 2b). In the MFM image the MNPs now appear as a depression, consistent with the concept of a particle that is magnetized by the magnetic stray field of the tip.^10^ As described above, the calibration requires determination of the maximum of the absolute value of the frequency shift Δ*f*_max_ for each particle. The original MFM image was filtered using a Wiener filter assuming Gaussian noise, which resulted in a significant improvement of the data. The region around the maximum of Δ*f* of the filtered signal was again fitted by a two-dimensional parabolic function to determine the maximum frequency shift Δ*f*_max_ of each MNP. The symmetry of the particles' MFM signals confirms the assumption that the magnetization of the MNPs is aligned by the stray field of the tip, that is, the magnetic anisotropy of the particles is negligible.

**Figure 3:**
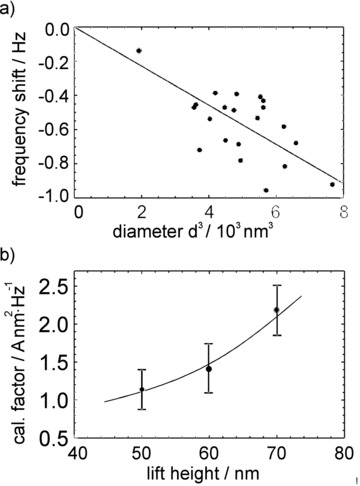
a) Plot of the calculated values of MFM signal versus the cubed diameter. The solid line shows a linear fit. b) Calibration factor as a function of the lift height; the error bars result from the error of the linear fit. The solid line is a guide to the eye.

In **Figure** **3**a the measured frequency shift of the 21 individual and undisturbed MNPs is plotted as a function of their cubed diameter. The displayed data show a clear linear dependence, thus underlining the feasibility of our calibration approach. The slope is determined from a linear fit with a fixed zero offset, according to the theoretical functional relation discussed above. The tip calibration factor *c*(*h*) then results as a product of the slope with the geometrical factor 6/π and the reciprocal saturation magnetization *M*_S_^−1^, *c*(*h*)^−1^ = Δ*f*·*d*^− 3^·6·π^−1^*M*_S_^−1^.

The resulting tip calibration factor is 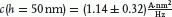. The indicated uncertainty results from the error of the linear fit and the uncertainty of *M*_S_.

To test the feasibility of our assumption of a negligible nonmagnetic shell, we also performed a linear regression with the offset as an additional fit parameter. In this case the resulting offset of the regression is (−0.09 ± 0.14) Hz. Hence it does not differ significantly from zero. These results justify the assumptions of a homogeneous magnetization in the MNPs and of a negligible nonmagnetic shell. The derived calibration factor *c*(*h*) relates the MFM signal for the given MFM tip and for the given lift height to the absolute value *m* of a magnetic moment of a specific MNP. Hence, the calibrated MFM tip operating at the same tip lift height *h* can be used to traceably measure the magnetic moment of any other spherical superparamagnetic MNP. Note that for traceable MFM measurements at different lift heights *h*, the tip calibration factor has to be determined again.

In Figure 3b the calibration factor *c*(*h*) derived for the same tip and three different lift heights of *h* = 50 nm, 60 nm, and 70 nm is plotted as a function of *h*. With increasing distance the sensitivity of the tip decays and therefore the calibration factor increases. The functional relation of the lift height dependence of the calibration factor results from a convolution of the decaying dipole field with the tip magnetization distribution and is not known a priori. Therefore, the line in Figure 3b serves as a guide to the eye.

Note that the analysis of the calibration factors as derived in this work is based on a relatively small number of particles. Therefore it is not known a priori that the size distribution of this selection of particles well mirrors the size distribution of the complete ensemble of MNPs as characterized by SQUID magnetometry and titration. However, the calibration scheme only relies on the assumption of a homogeneous and particle-independent magnetization, which is feasible due to the crystalline structure of the magnetite MNPs. The calibration scheme does not rely on the representative size distribution of the measured subensemble. Therefore, the calibration is not strongly dependent on the number of particles under consideration.

### 4.2. Quantitative Characterization of an MNP Sample

In the following, the calibrated tip characterized by the data of Figure 2 is used to characterize a different MNP sample prepared from the SHP20 suspension.

**Figure 4:**
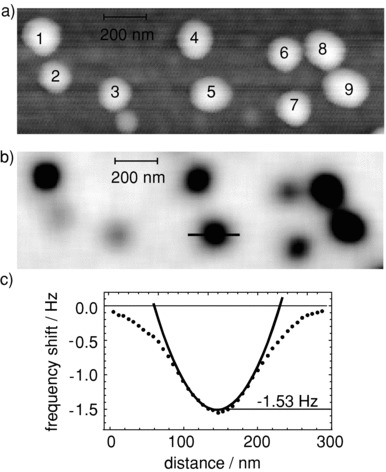
a) AFM and b) MFM images of MNPs measured with a calibrated tip at a lift height of 50 nm. c) The frequency shift Δ*f*_max_ was determined by parabolic fitting (solid line) to the experimental data above the center of the MNPs (dotted line), as exemplarily shown for particle #5. The magnetic moments for all MNPs are evaluated in SI units using the tip calibration factor and are summarized in Table 1.

**Figure** **4**a shows the AFM image of nine separated MNPs, numbered 1–9. The corresponding MFM image was measured at a lift height of 50 nm (Figure 4b). The maximum frequency shift Δ*f*_max_ of all particles was again derived from two-dimensional parabolic fits to the experimental data, exemplarily discussed for particle #5 (Figure 4c). From its maximum frequency shift, Δ*f*_max_ = (1.53 ± 0.1) Hz, the absolute value of the magnetic moment is determined using the tip calibration factor *c*(*h* = 50 nm) given above to be *m* = (1.74 ± 0.53) A nm^2^. The measurement uncertainty results from the uncertainty of the frequency measurement (i.e., the system noise that is estimated as 0.1 Hz after filtering) and the uncertainty of the calibration factor *c*. The magnetic moments of the other MNPs in Figure 4a were determined accordingly, and the results are summarized in **Table** **1**.

**Table 1:** Measured frequency shift and calculated magnetic moment of the particles in Figure 4a.

Particle no.	Frequency shift Δ*f* [Hz]	Magnetic moment [A nm^2^]
1	2.01 ± 0.1	2.30 ± 0.67
2	0.58 ± 0.1	0.65 ± 0.27
3	0.74 ± 0.1	0.84 ± 0.32
4	1.65 ± 0.1	1.84 ± 0.56
5	1.53 ± 0.1	1.74 ± 0.53
6	1.05 ± 0.1	1.20 ± 0.40
7	1.24 ± 0.1	1.42 ± 0.46
8	2.32 ± 0.1	2.65 ± 0.75
9	2.25 ± 0.1	2.57 ± 0.73

The described calibration procedure thus allows the traceable measurement of the magnetic moment of individual of superparamagnetic MNPs by MFM in SI Units.

## 5. Discussion

In the following, the limits of this new calibration technique are discussed. The calibration data show a certain straying around the linear fit. This can be mainly ascribed to noise in the measurement system. However, such deviations can also result from further apparatus deficiencies and from inappropriate nanoparticles. The first category covers a drift of the nominally constant lift height *h* due to piezo creep and piezo hysteresis during the MFM scan. Note, however, that the MFM instrument used in this work is operated with a closed-loop scanner to reduce the effect of piezo drift to the maximum extent. Furthermore, the MFM tip scans at a constant lift height *h* with respect to the substrate surface. However, the diameters and thereby the center positions of the measured particles vary causing a systematic error Δ*c* of the calibration factor *c*. This should result in an overestimation of Δ*f* for larger MNPs. However, such systematic deviation is not evident in the calibration curve and can thus be neglected.

Concerning the influence of the MNP properties on the calibration procedure, we assume a negligible anisotropy and a negligible nonmagnetic surface layer. When significant, both effects should be visible in the calibration curve. The effect of a nonmagnetic surface layer should lead to a significant zero offset of the linear fit, which is not present as discussed above. In contrast, the straying of the calibration data around the fit could be related to an effect of a non-negligible anisotropy stochastically inhibiting full alignment of **m** for all MNPs.

For our present measurement setup using a commercial instrument working in a self-excitation mode, the value of magnetic moment to be reliably resolved is limited by the resolution of our instrument. The noise level after filtering is less than 0.1 Hz. However, for frequency shifts smaller than about 0.4 Hz the fit of the shift does not reliably converge. Therefore, from this minimum Δ*f* of 0.4 Hz using the tip calibration factor 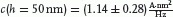, the minimum resolvable moment is estimated as *m*_min_ ≍ 0.5 A nm^2^. Smaller lift heights would mean higher magnetic sensitivity, however, and then the contribution of nonmagnetic interactions could gain importance.[9] For common tip geometries a larger tip volume and a higher magnetization of the tip coating would lead to a higher net magnetic moment of the tip, thereby enhancing the sensitivity of the technique as long as the perpendicular alignment of the tip magnetization can be maintained.

## 6. Conclusion

We have presented a technique for the traceable calibration of MFM tips that allows the quantitative measurement of the magnetic moments of individual MNPs in SI units. The resolution of the technique of 0.5 A nm^2^ is presently limited by the intrinsic noise of the MFM instrument employed and by the magnetic moment of the tip. The calibration scheme is versatile and can be transferred to practically any MFM setup for future application in nanomagnetism and biotechnology.

## 7. Experimental Section

*Prussian Blue Staining*: The iron concentration in the suspensions was estimated by Prussian blue staining in which Fe^3+^ ions, obtained by dissolution of the MNP suspension with hydrochloric acid and subsequent oxidation of Fe^2+^ with H_2_O_2_, form blue complexes with potassium ferrocyanide. Then the light absorption of the Prussian blue complexes was measured with a spectrophotometer at *λ* = 690 nm. Finally, the absorption value was related to the iron concentration via a calibration curve measured on samples with a known concentration of magnetite powder.
